# Supplementary material for: Drug Repurposing for Cystic Fibrosis: Identification of Drugs That Induce CFTR-Independent Fluid Secretion in Nasal Organoids
Source: Int J Mol Sci. 2022 Oct 21;23(20):12657. doi: 10.3390/ijms232012657 (PMC9603984; doi:10.3390/ijms232012657)

Supplemental figure S1

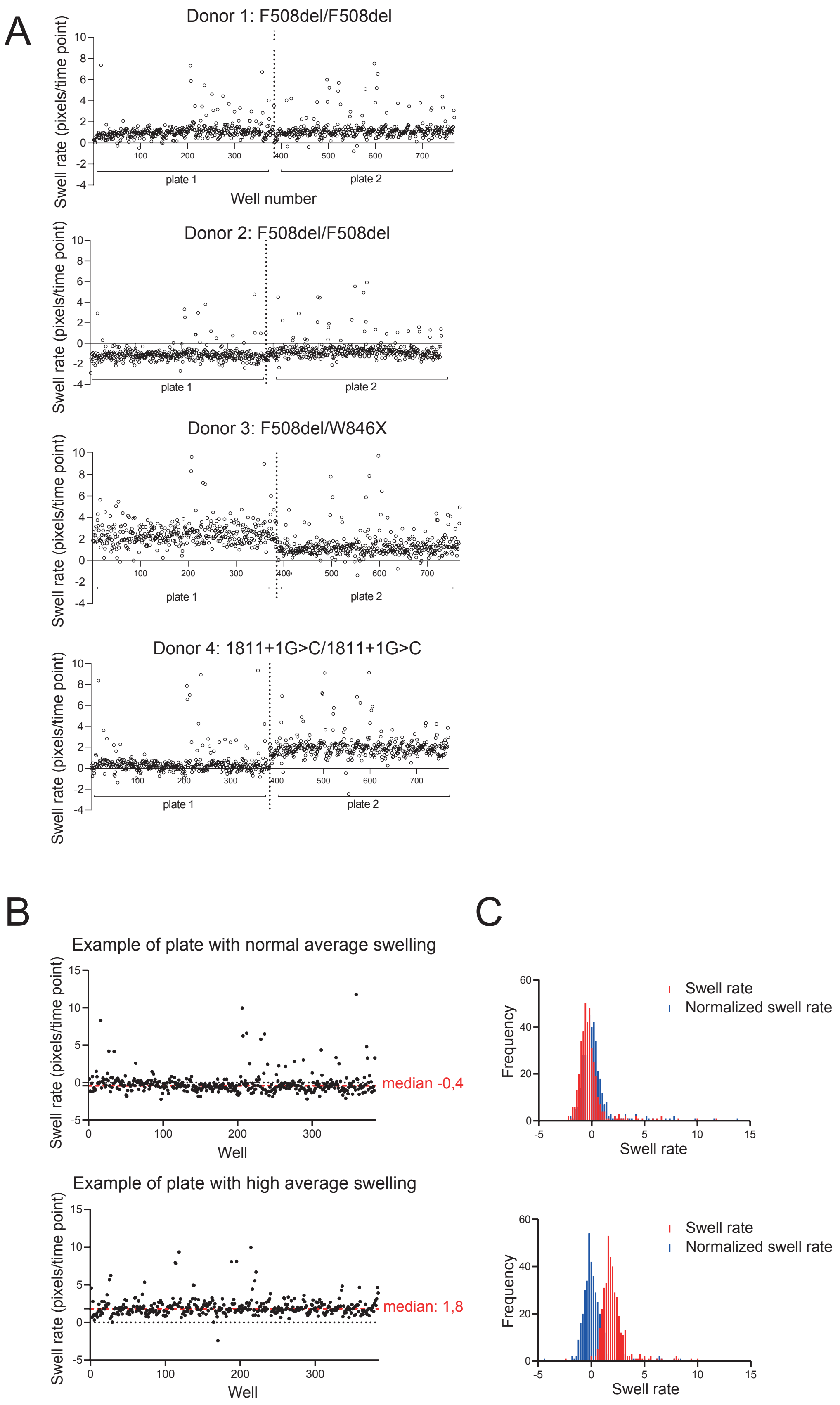

Supplemental figure S2

A

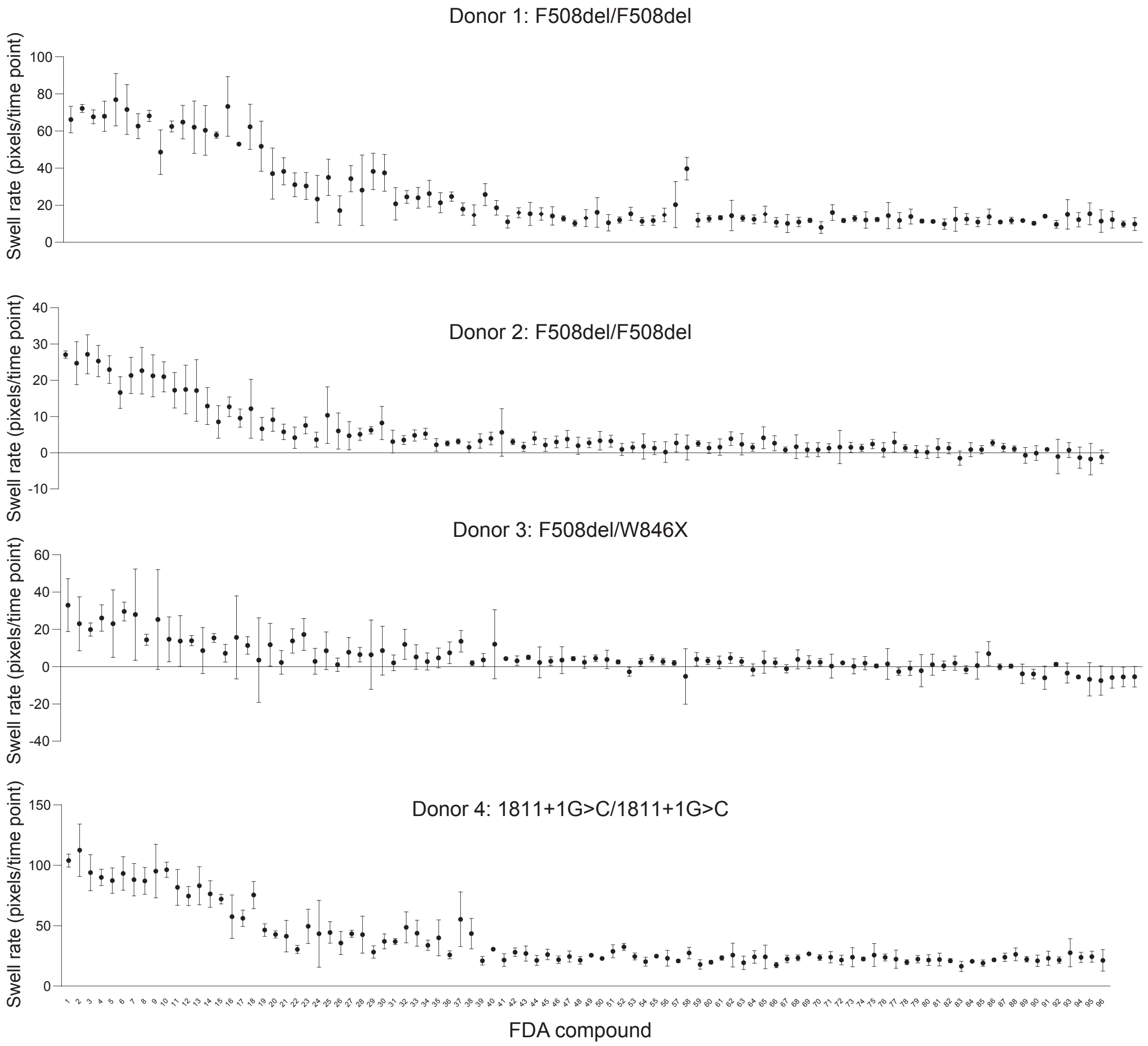

Supplemental figure S3

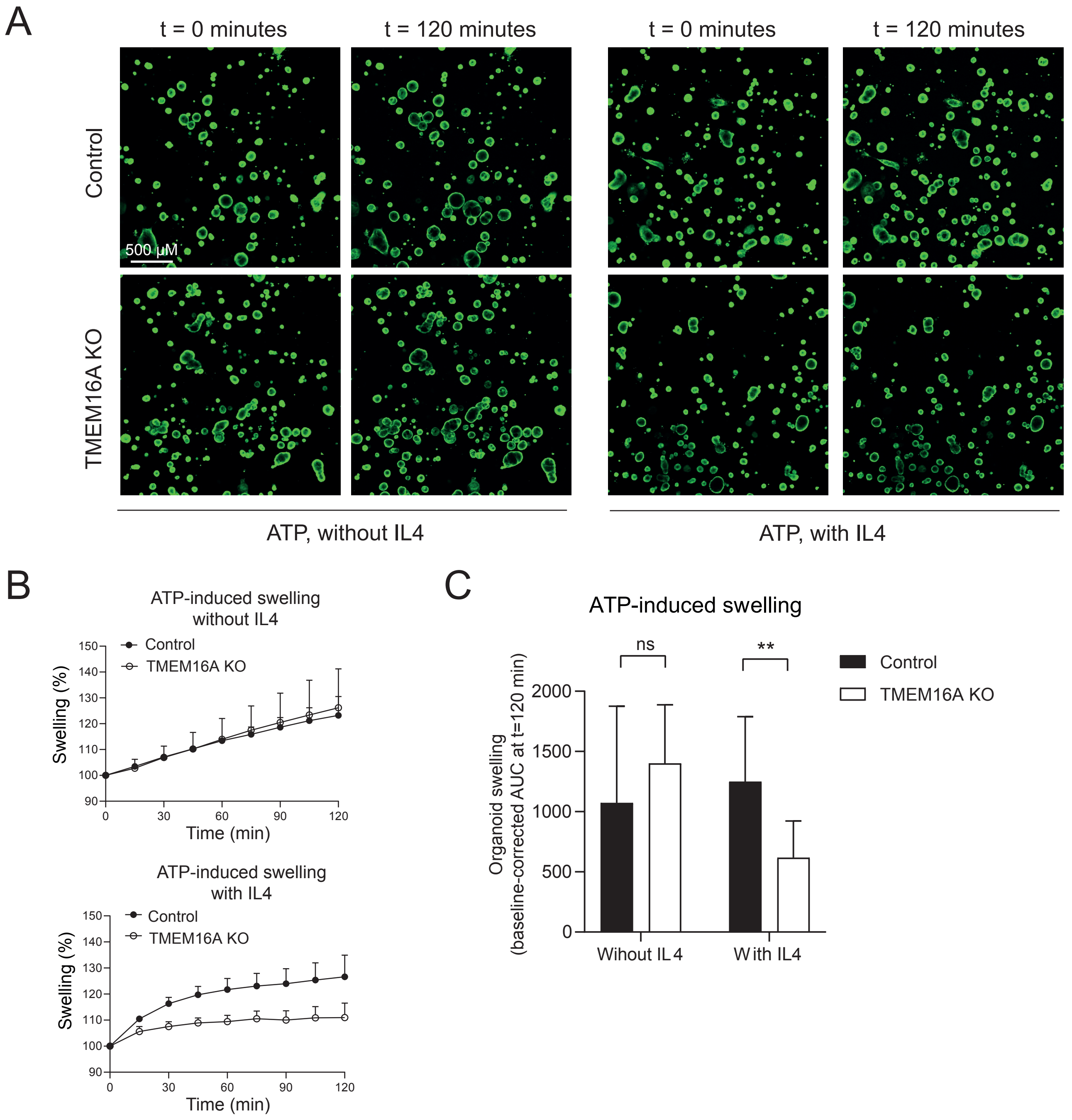

Supplemental figure S4

A

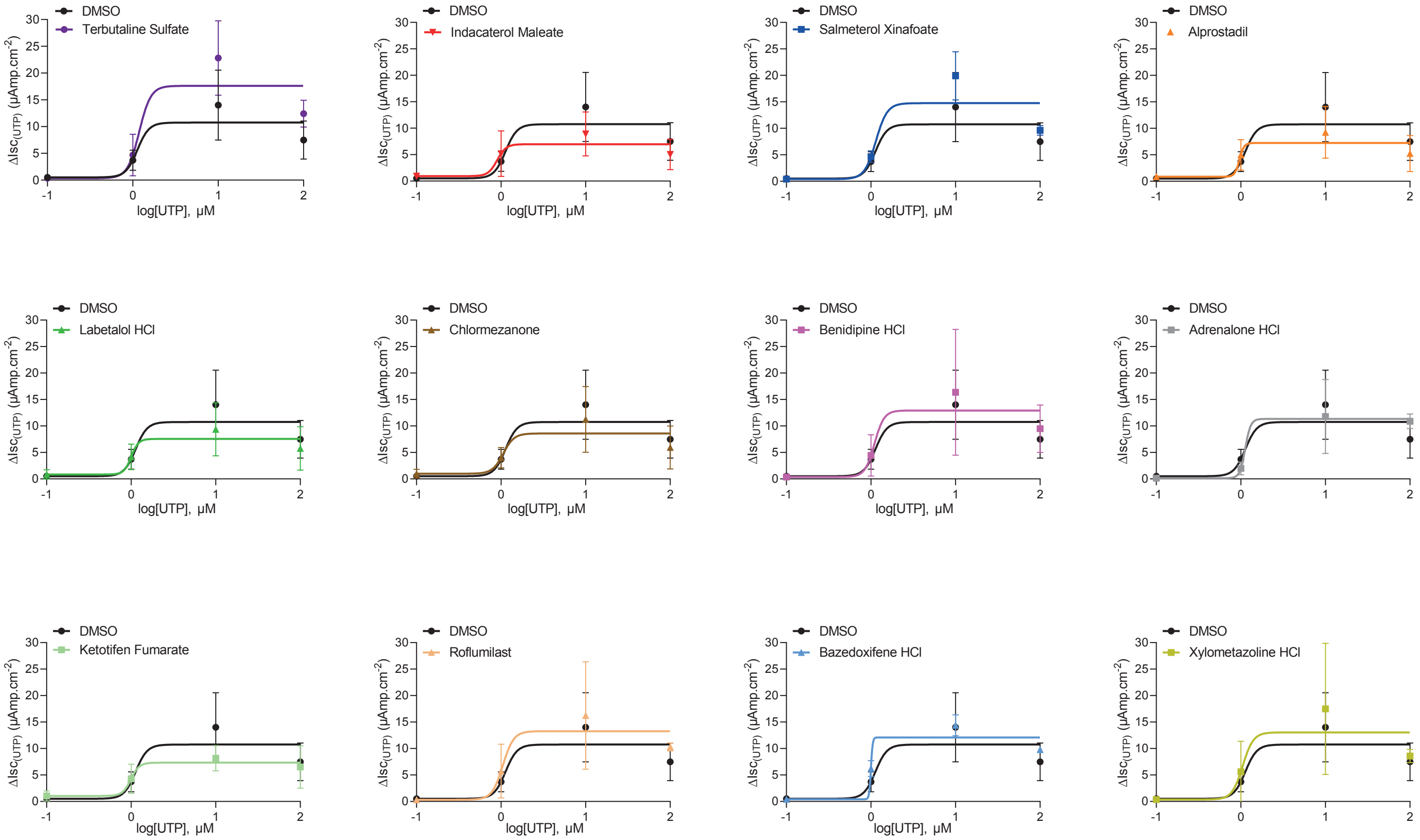

B

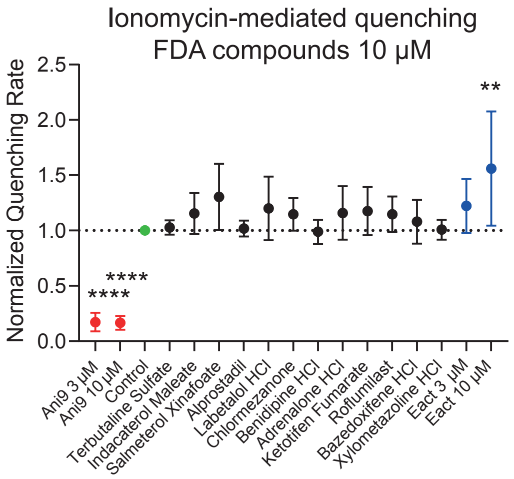

Supplement: Supplementary file 1 [file ijms-23-12657-s001.zip › 20220912 Supplementary_figures.pdf]
