# Supplementary material for: Drug Repurposing for Cystic Fibrosis: Identification of Drugs That Induce CFTR-Independent Fluid Secretion in Nasal Organoids
Source: Int J Mol Sci. 2022 Oct 21;23(20):12657. doi: 10.3390/ijms232012657 (PMC9603984; doi:10.3390/ijms232012657)
Supplement: Supplementary file 1 [file ijms-23-12657-s001.zip › Supplementary_figure_legends v1.pdf]

Figure S1. Primary screening assay in individual CF donors. (**a**) The primary screening assay was performed in four CF donors with different CFTR genotypes as indicated in the graphs (n=4 independent donors, 1-3 replicates per donor). Organoid swelling was determined in 384-wells plate format with 2 FDA compounds combined in a single well. Graphs depict the mean swell rate of all organoids in a well. Dotted lines represent different assay plates; (**b**) A plate normalization step was performed as median swell rates varied between assay plates and donors. Examples are shown from a plate with normal median swelling (upper panel) and from a plate with high median swelling (lower panel); (**c**) Histograms show the distribution of measurements before (red) and after (blue) plate normalization.

Figure S2. Secondary screening assay in individual CF donors. (**a**) The secondary screening assay was performed in the more conventional 96-wells plate format in the same four CF donors as the primary screening assay, as indicated in the graphs. Organoid swelling is shown for individual donors.

Figure S3. ATP-induced swelling in TMEM16A KO nasal organoids. (**a**) Representative confocal images (G542X/CFTRdele2.3(21kb)) of TMEM16A KO and control nasal organoids after stimulation with ATP. Organoids were treated with or without IL-4 for 48h; (**b,c**) Quantification of ATP-induced swelling in TMEM16A KO and control nasal organoids, treated without (upper panel) or with (lower panel) IL-4 for 48h (n=3 independent CFTR-null donors, 1-7 measurements per donor). Analysis of difference was performed using unpaired t-tests. ** $p < 0.01$.

Figure S4. Effect of the hit compounds on TMEM16A in other *in vitro* model systems. (**a**) The effect of hit compounds on UTP-dose response curves was analyzed in Ussing Chamber measurements with CFTR-null nasal cells. Data is shown for incubation with DMSO alone or with one of the 12 hit compounds. Nonlinear regression was used to fit a dose-response curve (n=3 independent donors, each compound was measured in at least 2 different donors; n =2-3 measurements per donor); (**b**) The Ionomycin-induced quenching assay in CFBE cells was also performed with a higher concentration of the FDA hit compounds (10 μM instead of 3 μM). TMEM16A-dependency was demonstrated with sensitivity for Ani9 (3 and 10 uM, shown in red). DMSO was used as negative control (shown in green) and E_{act} (3 and 10 uM) as positive control (shown in blue). For quantification, quenching rates were normalized to the control and a one-way ANOVA with Dunnett’s post-hoc test was used to analyze differences with control. ** $p < 0.01$, **** $p<0.0001$.
