# Supplementary material for: Drug Repurposing for Cystic Fibrosis: Identification of Drugs That Induce CFTR-Independent Fluid Secretion in Nasal Organoids
Source: Int J Mol Sci. 2022 Oct 21;23(20):12657. doi: 10.3390/ijms232012657 (PMC9603984; doi:10.3390/ijms232012657)
Supplement: Supplementary file 1 [file ijms-23-12657-s001.zip › Supplementary_tables v2.pdf]

Table S1. Basal cell isolation and expansion medium

Reagents	Concentration	Company
Bronchial epithelial cell medium-basal (BEpiCM-b)	50 % (v/v)	ScienCell, Carlsbad, CA, USA
Advanced DMEM/F12	23.5 % (v/v)	Gibco, Waltham, MA, USA
B-27 Supplement, serum free	2 % (v/v)	Gibco, Waltham, MA, USA
GlutaMAX Supplement	1 % (v/v)	Gibco, Waltham, MA, USA
HEPES (1 M)	10 mM	Gibco, Waltham, MA, USA
(±)-Epinephrine hydrochloride	0.5 µg/mL	Sigma-Aldrich, St Louis, MO, USA
Hydrocortisone	0.5 µg/mL	Sigma-Aldrich, St Louis, MO, USA
3,3′,5-Triiodo-L-thyronine sodium salt	100 nM	Sigma-Aldrich, St Louis, MO, USA
N-Acetyl-L-cysteine	1.25 mM	Sigma-Aldrich, St Louis, MO, USA
Nicotinamide	5 mM	Sigma-Aldrich, St Louis, MO, USA
SB 202190 (p38i)	500 nM	Sigma-Aldrich, St Louis, MO, USA
DMH-1 (BMPi)	1 µM	Selleck chemicals, Planegg, Germany
A83-01 (TGF-βi)	1 µM	Tocris, Bristol, UK
Y-27632 (ROCKi)	5 µM	Selleck chemicals, Planegg, Germany
DAPT (NOTCHi; only in expansion medium)	5 µg/mL	Fisher Scientific, Landsmeer, Netherlands
Recombinant human FGF-7	25 ng/mL	Peprotech, Rocky Hill, NJ, USA
Recombinant human FGF-10	100 ng/mL	Peprotech, Rocky Hill, NJ, USA
Recombinant human EGF	5 ng/mL	Peprotech, Rocky Hill, NJ, USA
Recombinant human HGF	25 ng/mL	Peprotech, Rocky Hill, NJ, USA
Rspondin 1 conditioned medium (from Rspo1 cells Cultrex®)	20 % (v/v)	Trevigen, Gaithersburg, MD, USA
Penicillin-Streptomycin	1 % (v/v)	Gibco, Waltham, MA, USA
Primocin	100 µg/mL	Invivogen, San Diego, CA, USA
Amphotericin B (only in isolation medium)	250 µg/mL	Gibco, Waltham, MA, USA
Gentamicin (only in isolation medium)	50 µg/mL	Sigma-Aldrich, St Louis, MO, USA
Vancomycin (only in isolation medium)	50 µg/mL	Sigma-Aldrich, St Louis, MO, USA

Table S2. ALI differentiation medium

Reagent	Concentration	Company
Advanced DMEM/F12	98.5% (v/v)	Gibco, Waltham, MA, USA
(±)-Epinephrine hydrochloride	0.5 µg/mL	Sigma-Aldrich, St Louis, MO, USA
Hydrocortisone	0.5 µg/mL	Sigma-Aldrich, St Louis, MO, USA
3,3′,5-Triiodo-L-thyronine sodium salt	100 nM	Sigma-Aldrich, St Louis, MO, USA
Penicillin-Streptomycin	1 % (v/v)	Gibco, Waltham, MA, USA
A83-01 (TGF-βi)	50 nM	Tocris, Bristol, UK
TTNPB (Retinoic acid agonist)	100 nM	Cayman, Ann Arbor, MI
Recombinant human EGF	0.5 ng/mL	Peprotech, Rocky Hill, NJ, USA

Table S3. Airway organoid medium

Reagent	Concentration	Company
Advanced DMEM/F12	95.5% (v/v)	Gibco, Waltham, MA, USA
B-27 Supplement, serum free	2 % (v/v)	Gibco, Waltham, MA, USA
GlutaMAX Supplement	1 % (v/v)	Gibco, Waltham, MA, USA
HEPES	10 mM	Gibco, Waltham, MA, USA
N-Acetyl-L-cysteine	1.25 mM	Sigma-Aldrich, St Louis, MO, USA
Nicotinamide	5 mM	Sigma-Aldrich, St Louis, MO, USA
SB 202190 (p38i)	500 nM	Sigma-Aldrich, St Louis, MO, USA
A83-01 (TGF-βi)	500 nM	Tocris, Bristol, UK
Y-27632 (ROCKi)	5 μM	Selleck chemicals, Planegg, Germany
Penicillin-Streptomycin	1 % (v/v)	Gibco, Waltham, MA, USA
Recombinant human FGF-7	5 ng/mL	Peprotech, Rocky Hill, NJ, USA
Recombinant human FGF-10	10 ng/mL	Peprotech, Rocky Hill, NJ, USA

Table S4. Antibodies

Antibody	Source	Identifier	Dilution
Mouse anti-MUC5AC	Thermo Fischer Scientific	#MA1-38223	1:500
Rabbit anti-β-tubulin IV	Abcam, Cambridge, UK	#ab179509	1:500
Rabbit anti-TMEM16A	Abcam, Cambridge, UK	AB64085	1:500
Rabbit anti-HSP90	Developed by laboratory of Prof. I. Braakman	NA	1:10.000
Goat anti-mouse IgG1, Alexa Fluor 647	Invitrogen, Waltham, MA, USA	A-21240	1:500
Goat anti-rabbit IgG, Alexa Fluor 488	Invitrogen, Waltham, MA, USA	A-11034	1:500
Goat anti-rabbit Immunoglobulins/HRP	Dako, Santa Clara, CA, USA	P0448;RRID:AB_2617138	1:2.000

Table S5. qPCR primers

Gene	Forward primer (5′- 3′)	Reverse primer (5′- 3′)
ANO1	AGGATTCCTTTTTCGACAGCAA	CGTTTTCACCGTTGTAGTCTCC
SLC26A9	GACTACATCATTCCTGACCTGC	AGGAGTAGAGGCCATTGACTG
SLC26A4	TGGTGGCTTGCAGATTGGAT	AGCTGTGAGACCAGCACTTG
CLCN2	TTGATCCTGCTCCCTTCCAG	CATAAGCATGGTCCACTCCC
SCNN1A	TCTGCACCTTTGGCATGATGT	GAAGACGAGCTTGTCCGAG
CFTR	CAACATCTAGTGAGCAGTCAGG	CCCAGGTAAGGGATGTATTGTG
ATP5B	TCACCCAGGCTGGTTCAGA	AGTGGCCAGGGTAGGCTGAT
RPL13A	AAGGTGGTGGTCGTACGCTGTG	CGGGAAGGGTTGGTGTTCATCC
